# Individualized plasticity autograft mimic with efficient bioactivity inducing osteogenesis

**DOI:** 10.1038/s41368-021-00120-w

**Published:** 2021-04-12

**Authors:** Yan Wei, Guixin Zhu, Zifan Zhao, Chengcheng Yin, Qin Zhao, Hudi Xu, Jinyang Wang, Jinglun Zhang, Xiaoxin Zhang, Yufeng Zhang, Haibin Xia

**Affiliations:** 1 The State Key Laboratory Breeding Base of Basic Science of Stomatology (Hubei-MOST) & Key Laboratory of Oral Biomedicine Ministry of Education, School & Hospital of Stomatology, Wuhan University, Wuhan, China; 2 Department of Oral Implantology, School and Hospital of Stomatology, Wuhan University, Wuhan, China

**Keywords:** Biomedical materials, Biomineralization

## Abstract

Mineralized tissue regeneration is an important and challenging part of the field of tissue engineering and regeneration. At present, autograft harvest procedures may cause secondary trauma to patients, while bone scaffold materials lack osteogenic activity, resulting in a limited application. Loaded with osteogenic induction growth factor can improve the osteoinductive performance of bone graft, but the explosive release of growth factor may also cause side effects. In this study, we innovatively used platelet-rich fibrin (PRF)-modified bone scaffolds (Bio-Oss^®^) to replace autograft, and used cytokine (BMP-2) to enhance osteogenesis. Encouragingly, this mixture, which we named “Autograft Mimic (AGM)”, has multiple functions and advantages. (1) The fiber network provided by PRF binds the entire bone scaffold together, thereby shaping the bone grafts and maintaining the space of the defect area. (2) The sustained release of BMP-2 from bone graft promoted bone regeneration continuously. (3) AGM recruited bone marrow mesenchymal stem cells (BMSCs) and promote their proliferation, migration, and osteogenic differentiation. Thus, AGM developed in this study can improve osteogenesis, and provide new guidance for the development of clinical bone grafts.

## Introduction

Since the twenty-first century, several studies have focused on the morphological recovery of tumors, infections, biochemical disorders, skeletal dysplasia, and trauma caused by extensive tissue defects^1,2^. Regeneration of hard tissues, including bone tissue, is important but challenging in tissue engineering and regeneration^3–5^. Autograft exhibits excellent regeneration effect, but the second surgery area increased the risk of postoperative complications^6^. Besides, the absorption of autograft is relatively fast and hard to control^7^. The autogenous bone is also difficult to adjust to the proper shape of the bone defect, which limits its application^8^. Therefore, researchers and clinicians set their sights on bone substitutes, which are widely used clinically because of their abundant sources^9^. However, bone substitutes lack biological components, such as osteoblasts and growth factors, resulting in slow bone regeneration and poor osteoinductivity^10^. Moreover, the granular morphology of bone substitutes is not conducive to clinical operations, causing certain difficulties to fit the defect area^11^. Although new bone regeneration materials are continuously emerging, ideal biological materials that can solve the abovementioned problems remain lacking.

The combination of osteogenic inductive growth factors can improve the osteoinductive properties of bone grafts and stimulate mesenchymal stem cells around the bone grafts to differentiate into chondroblasts or osteoblasts and form new bone^10^. Common growth factors include transforming growth factor-beta (TGF-β), bone morphogenetic proteins (BMPs), fibroblast growth factors, insulin-like growth factors (IGFs), and platelet-derived growth factors (PDGFs). These growth factors interact with each other during fracture healing and mediate bone regeneration^12–14^. BMP-2 is currently the only Food and Drug Administration (FDA)-approved osteoinductive growth factor, widely used with the bone substitutes in clinical practice^15^. BMP-2, an osteogenic cytokine, induces bone formation by promoting the proliferation and osteogenic differentiation of mesenchymal stem cells, which secret mineralized matrix and collagen locally^14,16,17^. BMP-2 combined with bone substitutes exerts osteoinductive effects, and bone substitutes serve as its carrier and scaffold to provide ideal mechanical properties for improved osteogenic effects^18–20^.

Although the combination of BMP-2 and bone substitutes has the above advantages in osteoinduction, BMP-2 is released explosively during the clinical application, and its half-life in vivo is short^21^. Excessive concentrations of BMP-2 in the early stages of mineralization may also cause side effects, such as ectopic osteogenesis, osteolysis and sedimentation, bone cysts, local inflammatory reactions, traumatic injuries, postoperative fever, and hemorrhage, and even cancer^15,22^. Therefore, the focus of our research is finding a carrier that can safely and effectively release BMP-2 and improve the operability of bone substitutes.

Platelet-rich fibrin (PRF), the second-generation biomaterial obtained by centrifugation from autologous blood, is rich in platelets and leukocytes, functions as a fibrin scaffold and regulator of various cytokines^23^. Numerous scholars have conducted in-depth research on PRF, confirming the positive role of PRF in the regeneration of soft tissue and hard tissue defects^24,25^. Micropores are composed of the fibers within PRF and can function as scaffolds for cell migration, proliferation, and differentiation as well as for delivery of growth factors^26^. Many studies have shown that PRF can gradually release its cytokines and cells^27,28^. It was shown that one of the reasons may be that cytokines are trapped in the fiber network, and even combine with the fiber and platelets in PRF^29^. With the fibrin network gradually destroy and remodel, the cytokines are released slowly. Also, PRF can protect growth factors from protease hydrolysis^30^. With the concept of low-speed centrifugation in recent years, modified liquid PRF demonstrates improved release of growth factors and abundance of leukocytes and platelets^23^. However, the endogenous growth facts release is far from enough to promote bone regeneration. Therefore, we supplemented FDA-approved BMP-2 to optimize the effect of bone regeneration^31,32^.

Here, we constructed an improved bone graft with osteoconductivity, osteoinductivity, enhanced mechanical properties, and good biocompatibility by using the bone substitute Bio-Oss^®^, the growth factor BMP-2, and liquid PRF. The new bone graft Bio-Oss^®^/PRF/BMP-2, named “autograft mimic (AGM)”, promoted the proliferation, migration, and mineralization of BMSCs in vitro and considerably improved bone regeneration in vivo. AGM overcomes most of the limitations of current bone substitutes and is capable of local sustained release of BMP-2 and its cytokines, thereby allowing BMP-2 to promote tissue regeneration continuously during the healing of bone defect and considerably enhance osteogenesis.

## Results

### Morphological characteristics of grafts

**Fig. 1:**
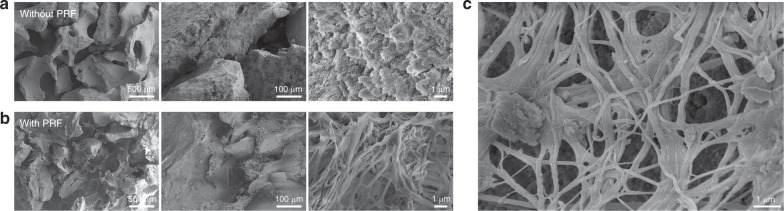
Fiber networks formed on the surface of Bio-Oss® to giving plasticity for AGM. **a**, **b** SEM micrographs of surface morphology of bone grafts with or without PRF. **c** SEM micrograph of the fibrin mesh between Bio-Oss® granules

Liquid PRF forms with lower centrifugal force and shorter centrifugal time than traditional PRF manufacturing methods^33^. The results suggest that PRF converts the morphology of Bio-Oss^®^ from granules into blocks and facilitates clinical operability (Fig. 1 and Supplement). Blood was collected into special plastic centrifuge tubes that do not contain anticoagulants. After centrifugation, the collected blood was divided into two layers: the upper PRF layer and the lower red blood cell layer (Supplement 1A). After mixing with PRF, Bio-Oss^®^ changed greatly from a conventional granular form into a block that can remains stable during operation (Supplement 1B). The morphology of the bone grafts was observed by scanning electron microscopy (SEM). Bio-Oss^®^ without PRF was dispersive at low-power objectives of ×40 (left) and ×200 (middle). When mixed with PRF, Bio-Oss^®^ was clustered with the filling of PRF. SEM under a high-power objective of ×10 000 (right) illustrated that the surface of Bio-Oss^®^ without PRF showed a typical hydroxyapatite structure, whereas that of Bio-Oss^®^ with PRF contained a large number of fiber bundles (Fig. 1a, b). And the fibrin mesh is obvious between Bio-Oss^®^ granules (Fig. 1c).

### Sustained release of BMP-2 in vitro

**Fig. 2:**
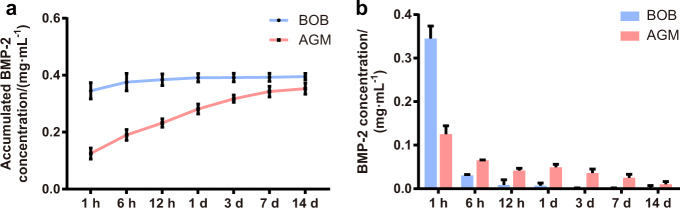
BMP-2 sustained release from AGM. **a** Total accumulated BMP-2 released from BOB and AGM over 14 days (*n* = 3). **b** BMP-2 release from BOB and AGM at each time point over 14 days (*n* = 3)

The three-dimensional (3D) network of fibers connecting bone substitutes was observed by SEM. Micropores composed of those fibers can serve as scaffolds for cell proliferation, migration, and differentiation likewise for delivery of growth factors^34^. Therefore, its controlled release characteristics were evaluated and quantified by measuring the accumulated BMP-2 concentration and the BMP-2 concentration at the required time point (Fig. 2a, b). In materials without PRF, the release of BMP-2 was explosive within the first 6 h, reached a peak quickly, and then almost stopped. (Fig. 2a). By contrast, in the grafts with PRF, the release of BMP-2 was more gradual and persistent, even maintained a small amount of release up to 14 days (Fig. 2a, b).

### AGM promotes BMSCs proliferation and migration in vitro

**Fig. 3:**
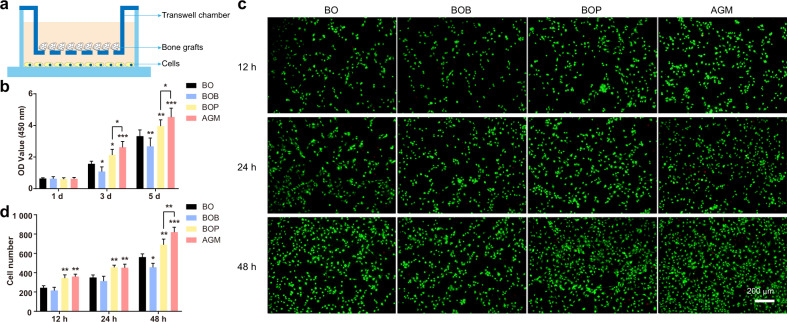
AGM have better biocompatibility. **a** Schematic diagram of cell culture strategy. **b** CCK-8 assay on cell proliferation at 1, 3, and 5 days. **c** Fluorescence microscopy images of BMSCs incubated with bone grafts after staining with Calcein-AM/PI dyes. **d** Quantification of living cell number in **c** (scale bar = 200 μm, *n* = 3, ***P < 0.001, **P < 0.01, *P < 0.05)

The effects of BMP-2 and PRF on cell proliferation and migration have been extensively studied in previous studies^35–38^. To further confirm the biological properties of the four different bone grafts, we co-cultured BMSCs harvested from a rat with the four samples and determined cell proliferation by the CCK-8 assay (Fig. 3a). Compared with the control group using Bio-Oss^®^ scaffolds alone, the BOP group significantly increased the number of cells on days 3 and 5. However, a significant decrease in proliferation ability was observed in the BOB group. Interestingly, there was a statistically significant increase in the proliferation of rat BMSCs in the AGM group compared with the other groups (Fig. 3b). We also performed living (green) and dead (red) cell fluorescence staining at different time points of co-culture and found that the number of living cells in the PRF-containing groups was significantly higher than that in the non-PRF-containing groups during the same period, which is consistent with the CCK-8 test results (Fig. 3c, d). Besides, only minimal dead and apoptotic cells were observed during the 5-day culture (Fig. 3c). These results suggest that PRF has good biocompatibility and can accelerate cell proliferation in the current culture system, whereas high concentrations of BMP-2 inhibited the proliferation of BMSCs. What is most important, AGM can retain BMP-2 in the fibrin network so that the BMP-2 can be released continuously for some time, thereby promoting cell proliferation.

**Fig. 4:**
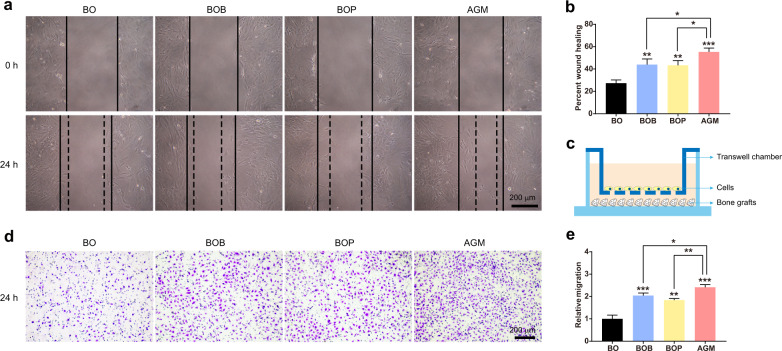
AGM has a stronger recruitment function on BMSCs. **a** Representative microscopic images of wound healing at the beginning and after incubated with bone grafts for 24 h. **b** Percent of wound healing was calculated as the ratio of the remaining scratch gap at 24 h and the original gap at 0 h. **c** Schematic diagram of cell culture strategy for transwell assay. **d** Representative microscopic images of BMSCs that migrated through the transwell in the migration assay. **e** The relative migration of cells compared to BO group in **d** (*n* = 3, ***P < 0.001, **P < 0.01, *P < 0.05)

Transwell migration assay and cell scratch wound-healing assay were used to determine the effects of four grafts on the chemotaxis of rat BMSCs. Compared with the control group, the cells in the BOB and the BOP groups migrated to the scratch area more. Besides, the cells migrated more significantly to the scratch area in the AGM group than in the BOB and BOP groups (Fig. 4a, b). Quantitative analysis of migrated cells using the transwell assay further confirmed the positive effects of AGM on cell migration (Fig. 4c–e).

### AGM promotes BMSCs mineralization in vitro

**Fig. 5:**
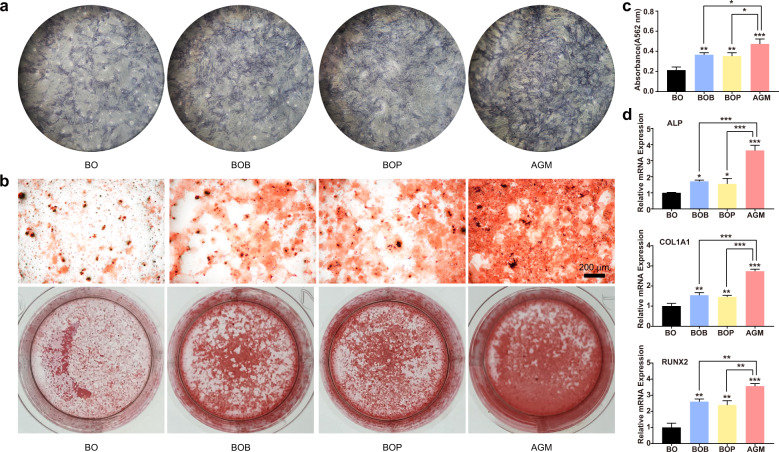
AGM has a stronger mineralization promotion effect on BMSCs. **a** ALP staining of BMSCs incubated with the corresponding bone grafts for 7 days. **b** ARS staining of BMSCs incubated with the corresponding bone grafts for 14 days. **c** Quantification of staining in **b**. **d** Osteogenic related genes (ALP, COL1A1, and RUNX2) expression were measured to confirm the osteoblast differentiation of BMSCs incubated with the corresponding bone grafts (*n* = 3, ***P < 0.001, **P < 0.01, *P < 0.05)

Alkaline phosphatase (ALP), a ubiquitous intracellular enzyme, is an indicator of the function and differentiation of osteoblasts; it is widely used in the study of abnormal bone metabolism and bone diseases and the detection of the degree of osteogenic differentiation^39^. To detect the osteogenesis effect of AGM in vitro, we assessed the ALP staining of rat BMSCs co-cultured with these four grafts on the 7th day of mineralization (Fig. 3a). Results showed that the three other groups showed better osteogenic effects than Bio-Oss^®^ alone, with the group treated with AGM providing the strongest osteogenic effect (Fig. 5a). Alizarin Red Staining and quantification showed the same results (Fig. 5b, c). Interestingly, PRF showed a similar strength of osteogenesis effect to BMP-2 possibly because of the release of endogenous growth factors in PRF^40^. To evaluate the changes of BMSCs at the gene level during osteogenesis induced by PRF and BMP-2, we measured the expression levels of osteogenic differentiation-related genes including ALP, COL1A1, and RUNX2. Results showed that both PRF and BMP-2 upregulated the expression of osteogenic differentiation-related genes, but no statistical difference was found between them, and the combined application could achieve a better osteogenesis effect (Fig. 5d).

### The regeneration effect of AGM in the bone defect in vivo

**Fig. 6:**
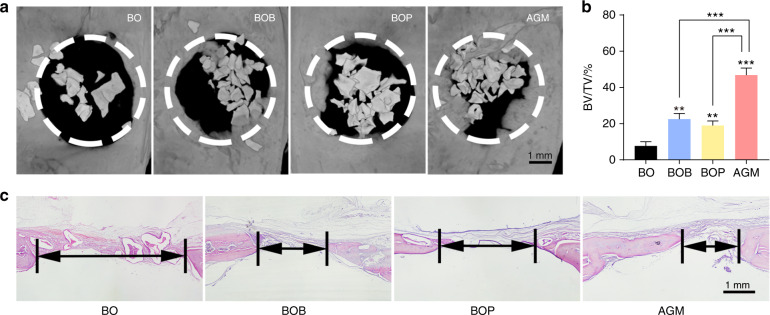
AGM has a better repair effect of critical parietal defects in rats. **a** Three-dimensional (3D) images showing the different repair results in parietal defects assessed by micro-CT. **b** Micro-CT analysis of BV/TV in parietal defects. **c** Representative HE staining images of parietal defects at 8 weeks postoperatively (scale bar = 1 mm, *n* = 3, ***P < 0.001, **P < 0.01)

**Fig. 7:**
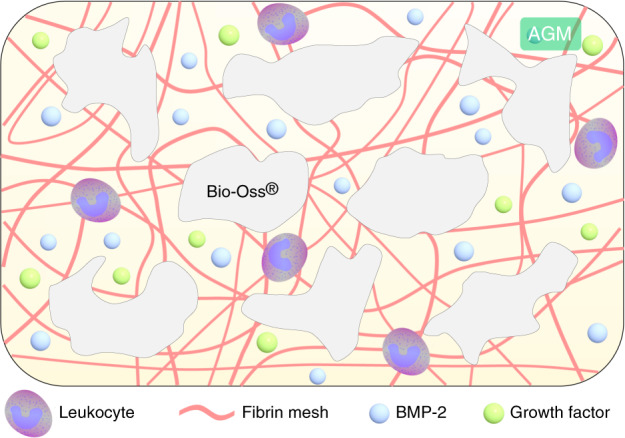
Schematic illustration of the AGM. First, the bone substitutes are agglomerated by PRF, allowing convenient operation and promoting graft stability after implantation. Secondly, AGM promotes the long-term and stable release of BMP-2. Next, the fibrin mesh within PRF can serve as a scaffold for cell migration, proliferation, and differentiation, providing a matrix for tissue reconstruction. Finally, leukocytes and growth factors secreted by PRF also play important roles in anti-infection and immunoregulation

To further confirm the osteogenesis ability of AGM in vivo, we prepared 5 mm critical-sized bone defects in the bilateral parietal of rats and repaired them using the corresponding bone grafts. After 8 weeks, micro-CT showed that a small new bone formation in the Bio-Oss^®^ group, whereas the other groups showed significant osteogenic effects (Fig. 6a). Notably, new bone formation was pronounced for AGM, with corresponding quantitative data showing a >40% ratio of bone to tissue in the defect area (Fig. 6b). Furthermore, bone substitutes moved around in the two groups without PRF (BO and BOB), whereas those in the other groups with PRF (BOP and AGM) were confined in the bone defect (Fig. 6a). Histological evaluation of HE staining can provide additional information about bone regeneration in the defect area (Fig. 6c). At 8 weeks after the surgery, HE staining showed that the tissue defect diameter was larger in the Bio-Oss^®^ group, whereas that in the three other groups, especially in AGM, was reduced to a certain extent, indicating rapid bone formation (Fig. 6c). These results suggest that PRF and BMP-2 exert synergistic effects on bone remodeling and new bone formation. And a hypothetical scheme of osteogenesis of AGM is proposed basing on the above results (Fig. 7).

## Discussion

Sustained release of growth factors to the surroundings is one of the goals of tissue engineering. In this study, bone grafts without PRF were found to release a high concentration of BMP-2 at an early stage, but this was not what we expected. On the one hand, fast-released growth factors are easily metabolized by the human body, preventing them from playing a long-term stabilizing role in promoting osteogenesis^21^. On the other hand, the explosive release of growth factors such as BMP-2 can cause high local concentrations, leading to side effects such as bone resorption, local inflammation, postoperative fever and bleeding, and even cancer^41,42^.

Unlike BOB which releases BMP-2 explosively, AGM releases BMP-2 slowly at a lower concentration. Studies have shown that high concentrations of BMP-2 can inhibit the proliferation of human primary periosteal cells, which plays an important role in fracture healing^38^. And lower concentrations of BMP-2 can promote the proliferation of chondrocytes^43^. Therefore, it is assumed that BOP explosively releases a high concentration of BMP-2 to inhibit cell proliferation. In contrast, with the sustained release of a low concentration of BMP-2 in AGM, the effect of promoting proliferation emerged. Besides, many studies have shown that BMP-2 composite delivery materials can promote the proliferation and osteogenic differentiation of osteoblasts at the same time^44–47^.

The famous “PASS” principle for successful guided bone regeneration surgery stands for space creation and maintenance, angiogenesis, primary wound closure, and stability of blood clots to allow regenerative tissue to grow in^48^. Therefore, bone grafts should be used for spatial maintenance during bone healing. However, in clinical treatment, the morphology of bone graft is hard to fit the defect unless titanium mesh or bone nails are used to fix the graft, leading to unstable osteogenic effect^49^.

Autografts are the gold standard for bone regeneration since they provide osteogenic cells, osteoinductive growth factors, and an osteoconductive scaffold, all essential for new bone formation^50,51^. However, autografts have brought several disadvantages because of the high absorptivity and complication. The AGM prepared by Bio-Oss^®^, PRF and BMP-2, was rich in bone substitutes with a low substitution rate, which could maintain the space of bone defect for a long time and play the role of osteoconduction. The fibrin scaffolds inside PRF wrapped around the bone graft, giving it initial stability similar to that of autograft. On the other hand, it has been reported that fibrin alone can play a role in provisional cell invasion and tissue regeneration^26^. Besides, integrated the effect of BMP-2, AGM simulated the characteristics of autograft in many aspects including osteoconduction and osteoinduction, which provided the potential possibility to mimic autograft in the clinic.

In the present study, we surprisingly discovered that xenograft modified by PRF has good operability and is easy to adjust to the shape of the bone defect. Heterogeneous bone grafts are of great clinical significance because they can prevent micromotion, minimize bone loss, and reduce the use of bone grafts and titanium meshes while maintaining space.

As mentioned above, Bio-Oss^®^, a commonly used bone scaffold material in clinical practice, plays an osteoconductive role and maintains the space of the defect area, while BMP-2 plays an osteoinductive role and promotes cell migration^21,37,52^. PRF extracted from blood is rich in various cells including platelets and a variety of cytokines, such as TGF-β1, PDGF, epidermal growth factor, and IGF, which play an important role in cell proliferation and migration^53^. PRF also contains a large number of leukocytes, which play a vital role in wound healing^54^. Leukocytes fight infection and regulate immunity by secreting key immunomodulatory cytokines such as interleukin-1β (IL-1β), IL-6, IL-4, and tumor necrosis factor-α to promote tissue healing^55–58^. The fibrin network in the PRF not only combines the entire bone scaffold to continuously release cytokines in the form of a hydrogel but also acts as a scaffold for cell migration, proliferation, and differentiation, providing a matrix for tissue reconstruction and accelerated mineralization^26,34^. In one-word, multiple factors of AGM jointly improve the effect of bone tissue regeneration.

Available bone grafts denote a wide range of materials, including allograft, xenograft, and artificial bone material^59^. In daily clinical applications, it is more than critical to select proper material depending on patient conditions. Implantation of the mix of autologous bone fragments and bone substitutes into the defect also showed an excellent osteogenic effect^60^. Drugs for topical application can also be personalized, and a variety of growth factors and cells can be used to promote osteogenesis and angiogenesis. In any case, it is crucial to combine bone substitutes and drugs through delivery materials. This study overcomes the secondary trauma of autologous transplantation, the inconvenience of xenotransplantation, and the explosive release of traditional BMP-2. Further, the bone graft proposed in this study partly compensates for the deficiencies of autograft and xenograft while maximizing their advantages, thereby providing practical guidance for the repair and treatment of clinical hard tissue defect. Moreover, this study not only fills the gap in current biological research of bone regeneration but also lays a solid theoretical foundation for the research and development of materials in tissue engineering and proposes new ideas for further development.

## Conclusions

In this study, BMP-2 and Bio-Oss^®^ were selected and improved by PRF, an autologous blood-derived preparation. The new type of bone graft, AGM, is clinically available and inherits the superior osteoconductivity property of Bio-Oss^®^ and osteoinduction property of BMP-2. It features strong operability and allows the controlled release of BMP-2. We confirmed the sustained release of BMP-2 in AGM, ensuring the long-term stable function of BMP-2. PRF and BMP-2 in AGM exerted synergistic effects in promoting the proliferation, migration, and osteogenic differentiation of BMSCs. Besides, the rat parietal defect model showed that BOB and BOP both improved the osteogenic performance of Bio-Oss^®^, and Bio-Oss^®^ combined with BMP-2 and PRF further improved the osteogenic performance in vivo. To a certain extent, these results support that AGM can solve the deficiencies of autograft and xenograft, and show great potential in clinical applications. Thus, this study lays the foundation for the future development of tissue engineering and provides new ideas for the modification of inorganic biomaterials.

## Materials and methods

### Preparation of PRF and bone grafts

For in vitro experiments, 8–10 mL of whole blood was obtained from the heart of a deeply anesthetized rat by cardiac puncture. The blood was then collected in the sterile vacuum tube (Kangjian Medical, Jiangsu, China) without anticoagulant and centrifuged immediately at room temperature at 700 r·min^–1^ for 3 min (IntraLock, USA). PRF, the upper plasma layer, was collected for subsequent experiments. For in vivo experiments, 2 mL of whole blood was extracted through the jugular vein to prepare PRF. The bone substitute (Bio-Oss^®^; Geistlich Biomaterials, Switzerland) and rat BMP-2 protein (Abbkine, California, USA) were used.

The experiment was divided into four groups: (1) Bio-Oss^®^ (BO): 0.25 g Bio-Oss_®_ + 0.2 mL phosphate-buffered saline (PBS), (2) Bio-Oss^®^/BMP-2 (BOB): 0.25 g Bio-Oss^®^ + 2 μg BMP-2 + 0.2 mL PBS, (3) Bio-Oss^®^/PRF (BOP): 0.25 g Bio-Oss^®^ + 0.2 mL PRF; and (4) Bio-Oss^®^/PRF/BMP-2 (AGM): 0.25 g Bio-Oss^®^ + 2 μg BMP-2 + 0.2 mL PRF.

### Scanning electron microscopy

The materials were fixed with 2.5% glutaraldehyde at 4 °C overnight. They were then rinsed thrice with PBS and double-distilled water (ddH_2_O); successively dehydrated once with 30%, 50%, 70%, 80%, and 95% ethanol; and then dehydrated twice with 100% ethanol for 5 min each. Critical point drying and spray gold treatment were performed. The cross-section of each sample was observed by SEM (Zeiss SIGMA, Carl Zeiss AG, UK).

### Release of BMP-2 in vitro

To determine the cumulative release of growth factors at 1 h, 6 h, 12 h, 1 d, 3 d, 7 d, and 14 d, we placed the samples in vials containing 5 mL of α-minimum essential medium (α-MEM) (HyClone, Thermo, USA) at 37 °C to release growth factors. At each time point, 0.5 mL of medium was collected and the same volume of α-MEM was supplemented. The release of BMP-2 was detected by enzyme-linked immunosorbent assay (ELISA) (Abcam, Cambridge, UK) according to the manufacturer’s instructions. Briefly, we coated the wells with standards and test samples, and blocked the remaining protein-binding sites in the coated wells with 5% non-fat dry milk. We added and incubated the following components in sequence: goat biotinylated detection polyclonal antibody against BMP-2, avidin-biotin-peroxidase complex, and TMB solution. After sufficient color development, we added stop solution to the wells and read the optical density at 450 nm. Finally, we prepared a standard curve and interpolated the concentration of test samples from this standard curve.

To determine the release of BMP-2 at 1 h, 6 h, 12 h, 1 d, 3 d, 7 d, and 14 d, at each time point, we placed the samples in vials containing 5 mL of α-MEM at 37 °C to release growth factors. At each time point, the culture solution was collected and 5 mL of fresh α-MEM was added. The release of BMP-2 was detected by ELISA (Abcam, Cambridge, UK).

### Cell culture and osteogenic induction

The transwell chamber (Corning, USA) with 3 μm pore membranes was selected for cell culture. The corresponding bone grafts were added in the upper chamber, and the lower chamber was used for cell culture. Rat BMSCs were harvested and incubated under standard cell culture conditions of 5% CO_2_ at 37 °C as previously reported^61^. The osteoblast inducing medium containing 50 μg·mL^–1^ ascorbic acid (Sigma-Aldrich, USA), 10 nM dexamethasone, and 10 mmol·L^–1^ β-glycerophosphate was used to replace the culture medium at 80% cell confluence. Cells were maintained under 5% CO_2_ at 37 °C with medium replacement every 3 days.

### Cell proliferation determination using the Cell Counting Kit-8 (CCK-8) assay

The proliferation of rat BMSCs was evaluated by the CCK-8 (Beyotime, China) after 1, 3, and 5 d of culture with the corresponding bone grafts. In brief, rat BMSCs were seeded into a 96-well plate at the density of 5 × 10^3^ cells per well. The solution containing 10 μL of CCK-8 original solution was used to replace the medium at 3 and 5 days. The cells were then incubated at 37 °C for 1 h. Absorbance at 450 nm was immediately quantified using a microplate reader (PowerWave XS2, BioTek, USA).

### Assessment of cell vitality in vitro

The cells were seeded into a 12-well plate with 3 × 10^5^ cells per well. After culturing in the incubator for 6 h, they were co-cultured with the corresponding bone grafts. Cells were collected at 12, 24, and 48 h for Calcein-AM/PI staining (YeaSen, Wuhan, China). Cells were first rinsed three times with 1× PBS and then incubated at 37 °C in the dark for 20 min with 500 μL of pre-prepared dye. Living/dead cells were detected and photographed under a fluorescence microscope using a 490 and 535 nm excitation filter, respectively (Olympus rCo, Japan).

### Cell scratch wound-healing assay

The cultured rat BMSCs were seeded in four six-well plates with 1 × 10^6^ cells per well. When the cells reached the exponential growth stage, the cell density was about 90%. A linear scratch was prepared in each well with a cell scraper, and these six-well plates were washed thrice with PBS to remove the subtracted cells. The cells were cultured with the corresponding culture medium in an incubator at 37 °C with 5% CO_2_. Sampling and taking photos were performed after culturing for 24 h.

### Transwell migration assays

Rat BMSCs were starved cultured with 2% fetal bovine serum (FBS) medium for 12 h and then seeded in a 12 mm transwell chamber with 1 × 10^4^ cells. The cells resuspended in α-MEM without FBS were added to the upper chamber, and each conventional culture medium containing the corresponding bone graft was added to the lower chamber. The cells were cultured in an incubator for 24 h and fixed in 4% formaldehyde for 15 min, stained with crystal violet (Beyotime, China), and washed with PBS three times before photographing.

### ALP staining and Alizarin red S (ARS) staining

The level of mineralization of the extracellular matrix was determined to ALP staining and ARS staining in vitro.

Rat BMMSCs were seeded in 24-well plates at a density of 1 × 10^5^ cells/well and cultured in an osteogenic medium. Rat BMSCs were washed with PBS thrice and fixed in 4% paraformaldehyde for 20 min at day 7 during the osteogenic differentiation. The cells were then stained using the ALP kit (Beyotime, China) following the manufacturer’s protocol. ARS staining was performed on day 14. Following 20 min of fixation in 4% paraformaldehyde, cells were washed thrice with ddH_2_O and then stained with 0.1% ARS staining solution (pH 4.5) for 10 min at 37 °C. Finally, the cells were rinsed gently with ddH_2_O to terminate the reaction. The mineralized nodules were photographed under an optical microscope. The nodules were dissolved by 10% cetylpyridinium chloride for 2 h. The absorbance at 562 nm represented the quantification of the ARS staining.

### Quantitative real-time PCR (RT-PCR)

Total RNA of BMSCs was isolated with Trizol reagent (Invitrogen, USA). Total RNA (1 μg) was used to synthesize complementary DNA using the PrimeScript RT reagent Kit (TaKaRa, Japan) as per the manufacturer’s instruction. The primer sequences used in the present study are listed in Table 1. The data were normalized to glyceraldehyde-3-phosphate dehydrogenase expression and analyzed using the comparison Ct (2^−ΔΔCt^) method.

**Table 1:** Primers used for real-time RT-PCR

Name	Direction	Sequence (5′–3′)
ALP	Forward	TGGACGGTGAACGGGAAAAT
	Reverse	TAGTTCTGCTCATGGACGCC
RUNX2	Forward	CCCAGTATGAGAGTAGGTGTCC
	Reverse	GGGTAAGACTGGTCATAGGACC
COL1A1	Forward	TGGCAAAGACGGACTCAAC
	Reverse	GGCAGGAAGCTGAAGTCATAA
GAPDH	Forward	GCACCGTCAAGGCTGAGAAC
	Reverse	TGGTGAAGACGCCAGTGGA

### Ethical approval and experiments in vivo

All rats utilized in the study were purchased from the Centre for Disease Control of the province of Hubei, China. The experiment was performed following the policies requirement of the Ethics Committee for Animal Research, Wuhan University, China. The study protocol was approved by the Ethics Committee for Animal Use of the Institute of Biomedical Sciences (Protocol number 69/2017). Female SD rats aged 8 weeks used a trephine drill with a diameter of 5 mm to prepare to penetrate critical-size defects in both parietal bones. Bio-Oss^®^ with different treatments was filled in the bone defects. After 8 weeks, the parietal bones were collected and fixed with 4% paraformaldehyde for 48 h at 4 °C for Micro-CT, and an indicator of bone volume/tissue volume (BV/TV) was determined to evaluate bone mass (SCANCO MEDICAL mCT50, Switzerland). The samples were processed by 10% ethylenediaminetetraacetic acid, which was changed every 3 days for 3 months for decalcification. The difference of newly formed bone among the four groups was compared using hematoxylin–eosin (HE) staining under the manufacturer’s direction after dehydration and paraffin embedding.

### Statistical analysis

Data analysis was performed using GraphPad Prism software 6.0. Differences between the two groups were evaluated through two-way ANOVA and Student’s *t*-test. All data are presented as mean ± SD. At least three repeats are contained for each experiment. Statistical significance was considered at *P < 0.05, **P < 0.01, ***P < 0.001.

## Supplementary information

Supplement 1

## Data Availability

Research data are not shared.
